# Precision of Pachymetry Measured With a Portable High‐Resolution Ultrasound, a Standard Ultrasound, and Two Scheimpflug Pachymeters

**DOI:** 10.1155/joph/4314297

**Published:** 2026-04-28

**Authors:** Anqi Liu, Youhan Ao, Yanyan Zhang, Zequan Xu, Feng Liu, Lina Mei, Mei Ge, Yifei Huang, Liqiang Wang

**Affiliations:** ^1^ Department of Ophthalmology, Chinese PLA General Hospital, No. 28 Fuxing Road Haidian District, Beijing, 100853, China, 301hospital.com.cn

**Keywords:** agreement, central corneal thickness, high-resolution ultrasound, pachymetry, repeatability, reproducibility

## Abstract

**Subject:**

To assess the precision (repeatability and reproducibility) and agreement of central corneal thickness (CCT) measurements by a high‐resolution ultrasound (E‐pach), a standard ultrasound, and two Scheimpflug pachymeters in healthy eyes.

**Methods:**

Instrument diagnostic test study. A total of 100 healthy volunteers were enrolled to measure right‐eye CCT by a high‐resolution ultrasound (E‐pach), a standard ultrasound (A‐scan device), and two Scheimpflug pachymeters (Pentacam and Corvis ST). To assess repeatability and reproducibility, the test−retest repeatability (TRT) and intraclass correlation coefficient (ICC) were calculated. The agreement among the four devices was evaluated with Bland−Altman plots.

**Results:**

The high‐resolution ultrasound (E‐pach) showed repeatability (ICC = 0.9981), interobserver reproducibility (ICC = 0.9971), and intersession reproducibility (ICC = 0.9825); the standard ultrasound and two Scheimpflug pachymeters also showed similar repeatability (all ICC ≥ 0.9679), interobserver reproducibility (all ICC ≥ 0.9730), and intersession reproducibility (all ICC ≥ 0.9647). However, the high‐resolution ultrasound yielded CCT values that were obviously lower than those of the standard ultrasound and Pentacam (*p* < 0.001) but higher than those of the Corvis ST (*p* < 0.001). The 95% limits of agreement (LoA) in the Bland−Altman plots were 44.5 µm (high‐resolution ultrasound vs. standard ultrasound), 34.9 µm (high‐resolution ultrasound vs. Corvis CT), and 32.5 µm (high‐resolution ultrasound vs. Pentacam).

**Conclusions:**

The high‐resolution ultrasound is a portable, reliable, and inexpensive pachymeter. However, the CCT values obtained from the high‐resolution ultrasound are not interchangeable with those from Pentacam, Corvis ST, and standard ultrasound.

## 1. Introduction

The accurate measurement of central corneal thickness (CCT) serves as a cornerstone in various ophthalmic practices, including the planning of refractive surgery [1, 2], the assessment of glaucoma [3–5], and the management of corneal diseases such as keratoconus and endothelial disorders [6–8]. Moreover, during the surgical procedure, it is crucial to enhance the precision of operations such as lamellar keratoplasty, particularly femtosecond laser−assisted keratoplasty. Femtosecond laser−assisted endothelial keratoplasty also requires accurate corneal thickness measurement [9, 10]. However, its practical application is often constrained by the limitations of existing technologies. While high‐precision imaging devices such as the Pentacam [11] and the Corvis ST [12] are capable of providing comprehensive corneal data and represent advanced technological standards, their reliance on sophisticated, stationary platforms makes them expensive and operationally complex. This inherent lack of portability creates a stark disparity in care quality, rendering these tools largely inaccessible in remote, resource‐limited, or intraoperative settings where real‐time feedback is critical.

The development of portable pachymeters, such as traditional handheld ultrasonic devices, aims to address this issue of accessibility [13]. Nevertheless, these portable solutions present their own set of limitations. For instance, the accuracy of devices such as the Pach‐Pen remains highly operator‐dependent and sensitive to probe alignment and contact pressure [14]. Meanwhile, the accuracy of portable optical devices, such as the Occuity PM1, in measuring edematous or scarred corneas remains unverified [15]. These persistent challenges underscore that portability alone is insufficient; the ideal device must also guarantee high accuracy and operational robustness across diverse clinical conditions. Therefore, a significant gap exists for a pachymeter that seamlessly combines the reliability of stationary gold‐standard devices with the practicality of a portable design.

The E‐pach, an A‐scan ultrasonic pachymeter, incorporates a high‐frequency transducer (> 50 MHz) for superior resolution and a unique algorithm that automatically selects optimal readings to minimize operator‐induced errors, thereby enhancing measurement objectivity. Furthermore, its handheld, lightweight design, coupled with a disinfectable ultrasound probe, makes it uniquely suitable for sterile surgical environments, enabling real‐time intraoperative assessment. However, despite these design innovations aimed at balancing portability with reliability, the precision of the E‐pach and its agreement with established gold‐standard devices remain unverified.

Therefore, to validate whether the portability of the E‐pach compromises its accuracy, this study aims to evaluate the precision (repeatability and reproducibility) of CCT measurements obtained with the E‐pach and compare its agreement with a standard ultrasound pachymeter and two types of stationary Scheimpflug imaging devices (Pentacam and Corvis ST).

## 2. Subjects and Methods

### 2.1. Subjects

This study was approved by the Office of Research Ethics at the General Hospital of People′s Liberation Army (PLA), and informed consent was acquired from all subjects. We enrolled healthy subjects recruited from a group of volunteers at the General Hospital of PLA from June 6th to July 6th.

In total, 100 healthy subjects were included in this study and met the following criteria: (1) communicated and cooperated well and (2) had satisfactory fixation ability in the right eye. The exclusion criteria of this study were active ocular pathology, corneal abnormal topographic patterns consistent with or suspected of keratoconus, any history of ocular surgery or trauma, recent contact lens wear (soft contact lens within two weeks and rigid contact lens within three months), systemic diseases with eye symptoms, and intraocular pressure > 21 mmHg.

### 2.2. Measurement Protocol

The measurement of precision and agreement strictly followed the British Standards Institute and the International Organization for Standardization (BSISO) [16].

All subjects underwent a complete ophthalmic examination, including uncorrected distance visual acuity, best corrected visual acuity, refraction, slit‐lamp microscopy, and fundus examinations; CCT was measured by the high‐resolution ultrasound (E‐pach; Sonogage Inc.), Pentacam, Corvis ST, and standard ultrasound. All measurements were performed from 8:00 to 10:00 or from 14:00 to 17:00 to minimize the diurnal variation in CCT readings.

In the first session, each subject had three consecutive measurements conducted by Observer A and Observer B to assess repeatability and interobserver reproducibility. In the second session (1 week later), each subject had an additional three consecutive scans by Observer A to assess intersession reproducibility.

To avoid any effect of the ultrasound probe and topical anesthetic on the cornea, the two noncontact pieces of equipment were used first. However, the sequence of measurements with the Pentacam and Corvis ST was randomly chosen, as well as with the standard ultrasound and high‐resolution ultrasound.

### 2.3. Instruments

**FIGURE 1:**
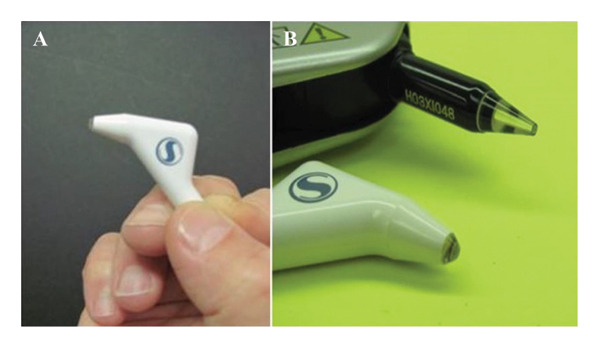
The E‐pach is a new, handheld ultrasonic pachymeter. (A) A handheld E‐pach. (B) The blunt tip of the probe to reduce corneal scratches.

The high‐resolution ultrasound E‐pach (Sonogage, Inc.) is a new, handheld, A‐scan ultrasonic pachymeter (Figure 1). Its transducer, of greater than 50 MHz, results in 2.5 times the accuracy of 20 MHz pachymeter transducers [17]. Measurements were taken at the central cornea. The subject’s head was stabilized with a chin rest, and they were instructed to look straight ahead and fixate on a distant target at eye level to maintain primary gaze. The probe was aligned perpendicular to the corneal surface using the pupil center as the target [18]. This procedure was performed by two independent operators to minimize potential bias related to corneal center localization. The E‐pach automatically acquired five measurements, with the minimum value among the 2nd to 4th readings recorded as the CCT. The reason it ignores the first and last readings is that one is coming on and off the cornea through the tear film, and the reason it chooses the smallest reading is that it is the most perpendicular value obtained. Moreover, the E‐pach can intraoperatively measure LASIK flaps, DSAEK tissue, and the residual stromal bed down to 50–60 microns.

The standard A‐scan ultrasound pachymeter (Pachette 500, DGH Technology Inc, Frazer, PA, USA) was used to evaluate CCT values. Before measurement, the cornea was anesthetised with topical 0.5% proparacaine hydrochloride (Alcaine; Alcon Laboratories, TX, USA).

The Pentacam is a widely used Scheimpflug photography, which is a noncontact pachymeter. Within 2 s, 25 slit images of the anterior segment are captured. For each slit image, mathematical software is used to detect edges, including the epithelium and endothelium of the cornea. The CCT value was only recorded when the correct alignment was obtained with the corneal apex.

Corvis ST is a relatively new Scheimpflug photography, which is also a noncontact tonometer equipped with an optical pachymetry function. For each measurement, the camera uses a sequence of 140 Scheimpflug images of the cornea. The device depicts the time required to applanate the cornea with an air puff, and the time of the first inward applanation is directly proportional to the IOP, which ranges from 1 to 60 mmHg. IOP and CCT are obtained during one measurement process.

### 2.4. Data and Statistical Analysis

All statistics were calculated using SPSS software for Windows Version 21 (SPSS Inc., Chicago, IL, US) and MedCalc Statistical Software Version 11.0 (MedCalc Software, Inc., Mariakerke, Belgium). The mean (±SD) for each common parameter from both devices was calculated. The repeatability, which equals the within‐subject (Sw) standard deviation, test−retest repeatability (TRT), and intraclass correlation coefficient (ICC), was calculated to assess precision (repeatability and interobserver and intersession reproducibility). The TRT was defined as 2.77 Sw, which represents the 95% confidence interval (CI) around the Sw within which 95% of measurements should occur. The ICC is a reliability coefficient that evaluates the consistency of datasets of repeated measurements and is between 0 and 1 (ICC ≤ 0.5, poor; 0.5 < ICC ≤ 0.75: moderate; 0.75 < ICC ≤ 0.90: good; and ICC > 0.9: excellent) [16, 19]. Bland−Altman plots were carried out to assess the agreement, which involves plotting the difference between the methods against their means. The 95% limits of agreement (LoA) were defined as ±1.96 standard deviations. Two instruments may be considered interchangeable if the 95% LoA is not clinically significant (10 µm).

## 3. Results

In this study, 100 healthy subjects (42 males) were enrolled. The mean age was 28.78 ± 4.01 years (range: 18–54).

### 3.1. Repeatability of Corneal Thickness Measurements

Table 1 displays the mean values, repeatability (Sw), TRT (2.77 Sw), and ICCs of three consecutive corneal thickness measurements made by Observer A and obtained by the four instruments. All ICCs were more than 0.96.

**TABLE 1:** The intraobserver repeatability of CCT measurements by different devices.

Instrument	Mean ± SD	Sw (D)	2.77 Sw (D)	ICC
E‐pach	545.0000 ± 33.5456	1.3360	3.7007	0.9981 (0.9973–0.9986)
A‐scan	567.50000 ± 34.8681	5.0666	14.0345	0.9797 (0.9718–0.9857)
Corvis ST	534.5000 ± 34.0839	5.4493	15.0946	0.9679 (0.9679–0.9837)
Pentacam	552.5000 ± 32.3173	4.1122	11.3908	0.9852 (0.9795–0.9896)

*Note:* Sw, within‐subject standard deviation; 2.77 Sw, test−retest repeatability (TRT).

Abbreviations: ICC, intraclass correlation coefficient; SD, standard deviation.

### 3.2. Interobserver Reproducibility of Corneal Thickness Measurements

Table 2 displays the mean values, repeatability (Sw), TRT (2.77 Sw), and ICCs of the corneal thickness measurements between the two observers obtained by the four instruments. All ICCs were more than 0.97.

**TABLE 2:** The intraobserver reproducibility of CCT measurements by different devices.

Instrument	Mean ± SD	Sw (D)	2.77 Sw (D)	ICC
E‐pach	536.7067 ± 33.7245	1.8080	5.0082	0.9971 (0.9957–0.9980)
A‐scan	560.6933 ± 33.8088	3.7720	10.4484	0.9879 (0.9820–0.9918)
Corvis ST	530.8417 ± 34.8129	5.6367	15.6137	0.9730 (0.9601–0.9817)
Pentacam	546.6967 ± 32.0052	3.5223	9.7568	0.9882 (0.9825–0.9921)

*Note:* Sw, within‐subject standard deviation; 2.77 Sw, test−retest repeatability (TRT).

Abbreviations: ICC, intraclass correlation coefficient; SD, standard deviation.

### 3.3. Intrasession Reproducibility of Corneal Thickness Measurements

Table 3 displays the mean values, repeatability (Sw), TRT (2.77 Sw), and ICCs for corneal thickness measurements between the two sessions (only by Observer A) obtained by the four instruments. All ICCs were more than 0.96.

**TABLE 3:** The intersession reproducibility of CCT measurements by different devices.

Instrument	Mean ± SD	Sw (D)	2.77 Sw (D)	ICC
E‐pach	535.8700 ± 32.8452	4.5166	12.5110	0.9825 (0.9742–0.9882)
A‐scan	559.7250 ± 34.4091	5.7353	15.8868	0.9733 (0.9606–0.9820)
Corvis ST	529.9867 ± 34.3264	6.4782	17.9446	0.9647 (0.9482–0.9761)
Pentacam	545.4884 ± 31.9253	5.0202	13.9060	0.9766 (0.9654–0.9842)

*Note:* Sw, within‐subject standard deviation; 2.77 Sw, test−retest repeatability (TRT).

Abbreviations: ICC, intraclass correlation coefficient; SD, standard deviation.

### 3.4. Comparison of Corneal Thickness Measurements Obtained by E‐Pach, A‐Scan, Corvis ST, and Pentacam

Table 4 displays the differences in corneal thickness measurements obtained from the four instruments.

**TABLE 4:** The agreement of CCT measurements by different devices.

Instrument	Mean difference ± SE	95% CI	*p* value
E‐pach vs. A‐scan	−23.797 ± 1.137	−26.052–−21.541	< 0.001
E‐pach vs. Corvis CT	5.997 ± 0.891	4.230–7.764	< 0.001
E‐pach vs. Pentacam	−9.577 ± 0.827	−11.218–−7.935	< 0.001
A‐scan vs. Corvis CT	29.793 ± 1.355	27.105–32.481	< 0.001
A‐scan vs. Pentacam	14.220 ± 1.278	11.684–16.756	< 0.001
Corvis CT vs. Pentacam	−15.573 ± 1.040	−17.636–−13.511	< 0.001

Abbreviations: CI, confidence interval; SE, standard error.

Corneal thickness values obtained with the high‐resolution ultrasound were statistically smaller than those obtained with the standard ultrasound (*p* < 0.001). Meanwhile, the 95% LoA in the Bland−Altman plots was 44.5 µm (Figure 2(a)).

FIGURE 2Bland−Altman plots of the mean central corneal thickness measurement against the differences in a comparison between E‐pach and A‐scan (a), E‐pach and Corvis ST (b), E‐pach and Pentacam (c), A‐scan and Corvis ST (d), A‐scan and Pentacam (e), and Corvis ST and Pentacam (f). The solid line indicates the mean difference. The interval between the upper and lower lines represents the 95% LoA.

**Figure:**
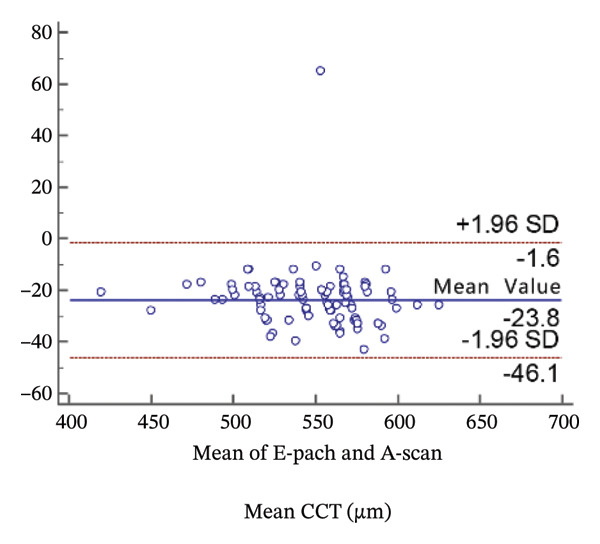
(a)

**Figure:**
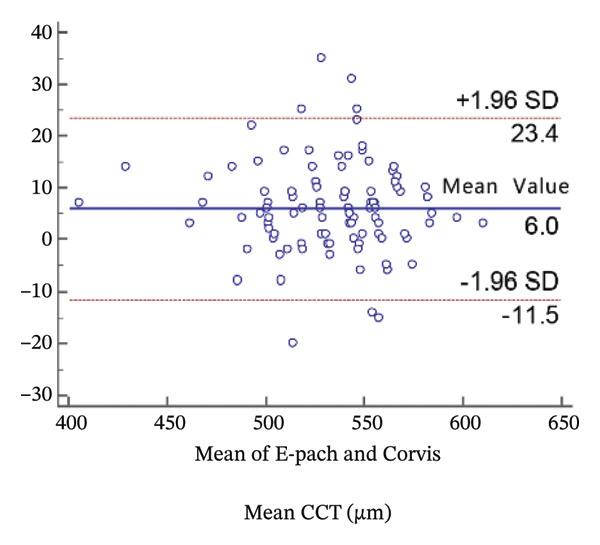
(b)

**Figure:**
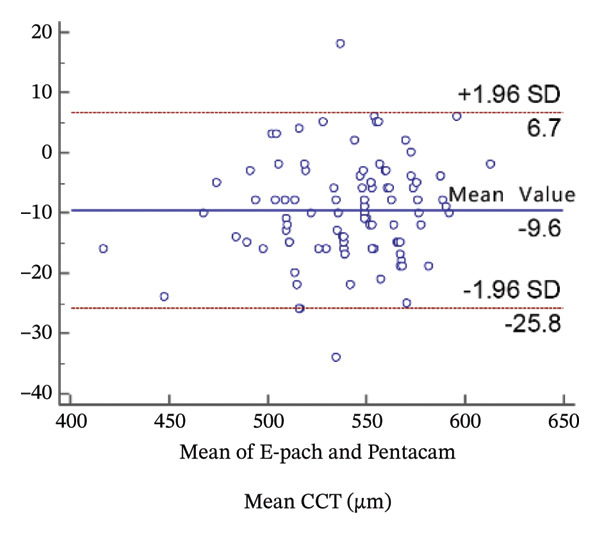
(c)

**Figure:**
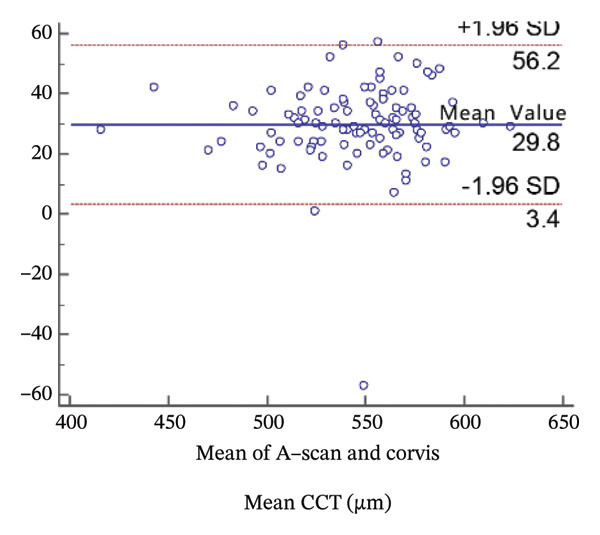
(d)

**Figure:**
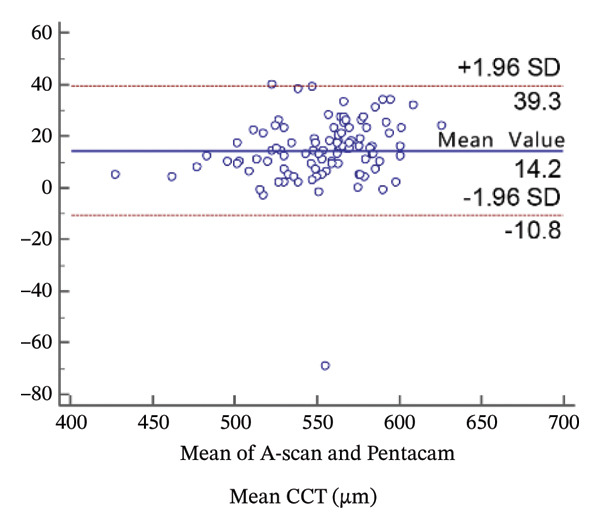
(e)

**Figure:**
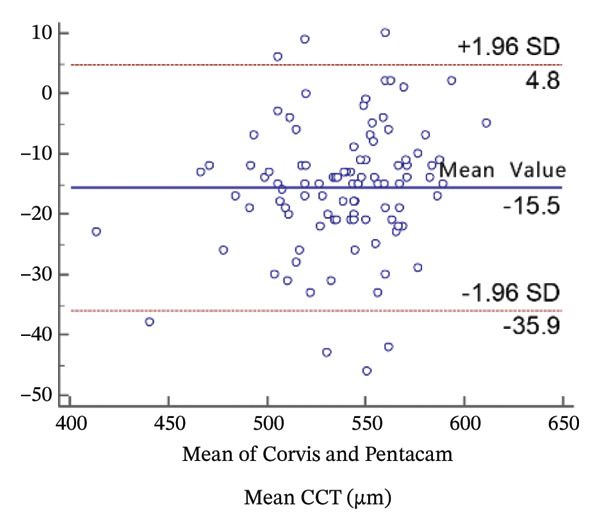
(f)

Corneal thickness values obtained with the high‐resolution ultrasound were statistically larger than those obtained with the Corvis CT (*p* < 0.001). Meanwhile, the 95% LoA in the Bland−Altman plots was 34.9 µm (Figure 2(b)).

Corneal thickness values obtained with the high‐resolution ultrasound were statistically smaller than those obtained with the Pentacam (*p* < 0.001). Meanwhile, the 95% LoA in the Bland−Altman plots was 32.5 µm (Figure 2(c)).

Corneal thickness values obtained with the standard ultrasound were statistically larger than those obtained with the Corvis CT (*p* < 0.001). Meanwhile, the 95% LoA in the Bland−Altman plots was 52.8 µm (Figure 2(d)).

Corneal thickness values obtained with the standard ultrasound were statistically larger than those obtained with the Pentacam (*p* < 0.001). Meanwhile, the 95% LoA in the Bland−Altman plots was 50.1 µm (Figure 2(e)).

Corneal thickness values obtained with the Corvis CT were statistically larger than those obtained with the Pentacam (*p* < 0.001). Meanwhile, the 95% LoA in the Bland−Altman plots was 40.7 µm (Figure 2(f)).

## 4. Discussion

The high‐resolution ultrasound is a new portable handheld pachymeter, and it can even measure intraoperatively DSAEK tissue and LASIK flaps down to 50–60 microns. However, no study has yet investigated the precision and agreement of this instrument. Our results demonstrated that it had great repeatability and reproducibility in CCT measurements. The agreement between the high‐resolution ultrasound and other instruments is, however, not good. The high‐resolution ultrasound yielded CCT values that were significantly lower than those from the standard ultrasound and Pentacam but significantly higher than those from the Corvis ST.

Our study showed great repeatability by the high‐resolution ultrasound: the TRT value was 3.7007 μm, and the ICC value was 0.9981 (far beyond 0.9). The repeatability of CCT values measured by other instruments was almost equally great. The repeatability of CCT values measured by the standard ultrasound, Corvis ST, and Pentacam devices in the present study is consistent with previous studies. Our study showed satisfying repeatability using the standard ultrasound: the TRT value was 14.0345 μm, and the ICC value was 0.9797. von Sonnleithner et al. [20] assessed the repeatability of CCT with the standard ultrasound. They found that the TRT value was 7.65 μm, and the ICC value was 0.994. Moreover, Liu et al. [17] also demonstrated that the ICC value of the standard ultrasound is 0.994. Our study also showed satisfying repeatability by Corvis ST: the TRT value was 15.0946 μm, and the ICC value was 0.9679. Yu et al. [21] and Chen et al. [22] both assessed the repeatability of CCT with the Corvis ST. They found that the TRT values were 13.0 μm and 12.56 μm, and the ICC values were 0.971 and 0.99. Lu et al. [23] reported an ICC of 0.979. Meanwhile, our study also showed satisfying repeatability using the Pentacam: the TRT value was 11.3098 μm, and the ICC value was 0.9852. Huang et al. [24], Viswanathan et al. [25], and Crawford et al. [26] evaluated the repeatability of CCT with Pentacam and indicated that the ICC values were 0.980, 0.984, and 0.979, respectively. The high‐resolution ultrasound made several attempts to improve the precision (repeatability and reproducibility) of CCT measurement. First, the E‐pach’s > 50 MHz transducer results in 2.5 times the accuracy of 20 MHz pachymeter transducers and 5 times the accuracy of 10 MHz transducers; second, it ignores the first and last readings because one is coming on and off the cornea through the tear film, and it minimizes the influence on the tear film.

In addition to repeatability, interobserver and intersession reproducibility of CCT measurements acquired with the abovementioned devices were also estimated. For interobserver reproducibility of the high‐resolution ultrasound, the TRT value was 5.0082 μm, and the ICC value was 0.9971; for intersession reproducibility, the TRT value was 12.5110 μm, and the ICC value was 0.9825. As expected, the intersession reproducibility was slightly worse than the interobserver reproducibility; however, the general reproducibility was far beyond good (ICC = 0.90). The reproducibility of CCT values measured by the standard ultrasound, Corvis ST, and Pentacam devices was almost equally great. The reproducibility of CCT values measured by the standard ultrasound, Corvis ST, and Pentacam devices in the present study was similar to previous studies. Nam et al. [27] evaluated the interobserver reproducibility of CCT measurements using A‐scan. They found a TRT value of 5.2 μm and an ICC value of 0.995. Ali et al. [28] evaluated the intersession reproducibility of CCT measurement using Corvis ST. They found a TRT value of 11 μm and an ICC value of 0.980. Bourges et al. [29] assessed the interobserver and intersession reproducibility using Pentacam at ICC values of 0.993 and 0.980, respectively.

When the CCT readings were compared among the four instruments, a significant difference in average CCT values was observed. Specifically, in the results of the *t*‐test, average CCT values of the standard ultrasound were significantly higher than those of the Pentacam; however, these Pentacam values were significantly higher than those of the high‐resolution ultrasound, which were significantly higher than those of the Corvis CT. The results of the Bland−Altman plots were consistent with the *t*‐test.

Several recent studies have reported that CCT readings of the standard ultrasound are higher than those of the Pentacam and Corvis ST devices. Smedowski et al. [30] and Gokcinar et al. [19] both assessed the agreement of CCT readings using the Pentacam, Corvis ST, and A‐scan devices. They both indicated that the mean CCT value was the highest for the A‐scan and the lowest for the Corvis ST, which was similar to our results. Other studies also reported a small underestimation of CCT values with the Pentacam [31–35] or Corvis CT [36] compared to the standard ultrasound. Similarly, a recent study reported that smaller values were obtained with the other Scheimpflug Pachymeter [19, 26, 35, 37, 38] compared to the standard ultrasound.

There are two possible explanations for this underestimation: (1) the CCT readings by the standard ultrasound were affected after giving topical 0.5% proparacaine hydrochloride, which increased corneal thickness (8.6 μm increase in 80 s) [39] and (2) the accuracy of the standard ultrasound is influenced by whether the probe is placed as perpendicular as possible to the center of the cornea [40–42]. The results, however, are still controversial. For example, Tai et al. [43] showed that the CCT value of the Pentacam is overestimated by 10 μm compared to that of the A‐scan. A possible reason could be compression of the cornea or displacement of the tear film when measuring by ultrasound, which may yield slightly thinner readings.

Moreover, multiple factors can affect the accuracy of CCT measurements. Wang’s study indicated that the agreement of CCT measurements is influenced by the corneal thickness itself; although parameters measured by Corvis ST demonstrate good overall repeatability, the degree of corneal deformation is correlated with corneal thickness. The agreement in CCT measurements between Corvis ST and either Pentacam HR or OCT is poor. These differences are too large to be considered clinically interchangeable [44]. In addition, a higher corneal refractive index tends to cause Scheimpflug‐based devices to underestimate CCT, whereas optical coherence tomography (OCT) tends to overestimate it [45].

In our study, it is worth mentioning that the advantages of E‐pach lie not only in its excellent repeatability and reproducibility but also in the enhanced precision afforded by its > 50 MHz high‐frequency ultrasound transducer. Its handheld, lightweight, and portable design makes it more suitable for surgical settings compared to noncontact devices, enabling real‐time intraoperative measurement of corneal thickness and graft apposition, thereby providing immediate feedback to guide surgical decisions. Moreover, CCT readings of the E‐pach are significantly lower than those of the standard ultrasound, even lower than Pentacam. The possible explanation could be that high‐resolution ultrasound chooses the smallest of the middle three readings out of five measurements by ignoring the first and last readings. To ignore the first and last readings (measuring when coming on and off the cornea through tear film), E‐pach minimizes the influence of tear film; to choose the smallest reading, E‐pach obtained the most perpendicular value of CCT measurement, which is the closest value to CCT, thereby minimizing operator‐induced errors.

Although the CCT readings of the high‐resolution ultrasound were statistically and clinically different (> 10 µm) from those of the other instruments (whether by *t*‐test or by Bland−Altman), the E‐pach is not interchangeable with them, and its high precision, portability, and real‐time capability make it a reliable measurement option in specialized clinical scenarios, particularly during surgery or in settings where conventional devices are impractical.

There were some limitations in our present study. We only evaluated the precision and agreement of CCT measurements in normal eyes. In further research, ocular disorders including glaucoma, myopia, keratoconus, or postrefractive surgery could be included. In addition, other types of instruments could be included in future studies.

## 5. Conclusion

The new versions of the high‐resolution ultrasound (E‐pach) are portable, relatively inexpensive, and reliable (our study indicated that it displayed great repeatability and reproducibility). However, the CCT values obtained from the high‐resolution ultrasound are not interchangeable with those from the standard ultrasound, Pentacam, and Corvis ST devices.

**Nomenclature**
CCT: Central corneal thickness
USP: Ultrasound pachymetry
TRT: Test−retest repeatability
ICC: Intraclass correlation coefficient
BSISO: British Standards Institute and the International Organization for Standardization
CI: Confidence interval
LoA: Limits of agreement

## Author Contributions

Conceived and designed the experiments: Zequan Xu and Liqiang Wang. Performed the experiments: Anqi Liu and Feng Liu. Analyzed the data: Youhan Ao, Yanyan Zhang, and Zequan Xu. Contributed reagents/materials/analysis tools: Lina Mei and Mei Ge. Wrote the paper: Anqi Liu. Critical revision of the manuscript: Liqiang Wang and Yifei Huang.

## Funding

This work was carried out without any external financial support.

## Disclosure

A pre‐print version of this study has been previously published on Research Square (Liu et al., [17]) and is cited accordingly.

## Ethics Statement

The Declaration of Helsinki was strictly followed in all procedures. Written informed consent was obtained from all subjects. The study was approved by the Medical Ethics Committee of the General Hospital of the People’s Liberation Army (PLA).

## Consent

Please see the Ethics Statement.

## Conflicts of Interest

The authors declare no conflicts of interest.

## Data Availability

The data that support the findings of this study are available from the corresponding author upon reasonable request.
